# Sodium Salicylate Reduced Insulin Resistance in the Retina of a Type 2 Diabetic Rat Model

**DOI:** 10.1371/journal.pone.0125505

**Published:** 2015-04-14

**Authors:** Youde Jiang, Shalini Thakran, Rajini Bheemreddy, William Coppess, Robert J. Walker, Jena J. Steinle

**Affiliations:** 1 Department of Anatomy and Cell Biology, Wayne State University, Detroit, Michigan, United States of America; 2 Department of Ophthalmology, Wayne State University, Detroit, Michigan, United States of America; 3 Department of Ophthalmology, University of Tennessee Health Science Center, Memphis, Tennessee, United States of America; 4 VA Medical Center, Memphis, Tennessee, United States of America; 5 Philder Smith College, Little Rock, Arkansas, United States of America; Children's Hospital Boston, UNITED STATES

## Abstract

Sodium salicylate has been reported to reduce markers of diabetic retinopathy in a type 1 rat model. Because rates of type 2 diabetes are on the rise, we wanted to determine whether salicylate could improve insulin resistance in a type 2 rat model, as well as improve retinal function. We treated lean and obese BBZDR/Wor type 2 diabetic rats with salicylate in their chow for 2 months. Prior to salicylate treatment, rats underwent an electroretinogram to measure retinal function. After 2 months of treatment, rats underwent an additional electroretinogram prior to sacrifice. In addition to the animal model, we also treated retinal endothelial cells (REC) and rat Müller cells with salicylate and performed the same analyses as done for the rat retinal lysates. To investigate the role of salicylate in insulin signaling, we measured TNFα and caspase 3 levels by ELISA, as well as performed Western blotting for insulin receptor substrate 1, insulin receptor, SOCS3, and pro- and anti-apoptotic markers. Data demonstrated that salicylate significantly improved retinal function, as well as reduced TNFα and SOCS3-induced insulin resistance in all samples. Overall, results suggest that salicylate is effective in reducing insulin resistance in the retina of type 2 diabetic rat models.

## Introduction

With current rates of diabetes continuing to skyrocket, increased understanding of insulin signaling both systemically and in specific organs becomes critical. Diabetic retinopathy is the leading cause of vision loss in working age adults, with 28.5% of people over 40 having some retinal changes indicative of diabetic retinopathy (statistics from 2005–2008, American Diabetic Association). To best treat patients with diabetic retinopathy, improved understanding of the retinal changes in response to dysfunctional insulin signaling becomes increasingly critical.

One factor that is potentially involved in the regulation of insulin signaling is increased tumor necrosis factor alpha (TNFα) levels associated with hyperglycemia [1]. We have previously reported that high glucose leads to increased TNFα levels in whole retina of diabetic rats [2], as well as in retinal endothelial cells (REC) [3] and Müller cells [4]. Increased TNFα can disrupt insulin signaling in a number of ways, but most work in adipocytes and embryo fibroblasts suggests that TNFα leads to a preferential phosphorylation on insulin receptor substrate 1 (IRS-1) on serine 307 (serine 312 in humans), which disrupts insulin signaling to Akt, a key anti-apoptotic protein activated by insulin [5,6]. We have shown similar results in retinal endothelial cells [3]. In addition to TNFα actions on IRS-1, TNFα also can activate other proteins known to be involved in insulin resistance, namely suppressor of cytokine signaling 3 (SOCS3) [7,8]. Activation of SOCS3 leads to phosphorylation of the insulin receptor on tyrosine 960, which blocks the interaction of insulin receptor and IRS-1, leading to obstruction of insulin receptor signaling [8]. Therefore, inhibition of TNFα actions in response to hyperglycemia would likely eliminate insulin resistance through multiple pathways.

One pathway of interest is through decreasing TNFα-mediated activation of I-kappa B kinase beta (IKKα). Inhibition of IkB allows TNFα to activate nuclear factor kappa B (NFkB), which is associated with impaired insulin signaling [9,10]. One therapy that has been shown to decrease retinal markers of diabetic retinopathy in rodents through inhibition of the IKKβ pathway is sodium salicylate [11]. Others have also reported that salicylate is also effective as a therapy in Parkinson’s disease due to its actions in IKKβ inhibition, leading to lowered TNFα levels [12]. Additionally, salicylate has been reported to reduce insulin resistance in human umbilical vein endothelial cells [13] and in the liver of Wistar rats fed a high fatty acid diet [14] via reduced IKKβ and TNFα. While salicylate appears to be beneficial in a number of models, others have reported that salicylate can induce apoptosis. Work in HCT116 colorectal cancer cells treated with salicylate showed increased levels of Fas ligand and Bcl-2 family proteins [15]. Others have reported that salicylate leads to caspase 3 activation and apoptosis in guinea pig cochlea [16]. Therefore, it is clear that salicylate reduces IKKβ levels, but the downstream effects of this inhibition appear to be tissue/organ specific.

Because others have reported that sodium salicylate is effective in reducing neuronal thickness and vascular changes associated with diabetic retinopathy in a type 1 rat model [11], we wanted to confirm whether salicylate also improved insulin signaling in a type 2 diabetic rat model. Additionally, we wanted to test the effects of salicylate therapy on insulin signaling cascades on 2 key retinal cell types involved in diabetic retinopathy, the Müller cell and retinal endothelial cell. We hypothesized that sodium salicylate would reduce IKKβ, leading to reduced TNFα and improved insulin signaling in the BBZDR/Wor obese rat model of type 2 diabetes. We also investigated the actions of sodium salicylate on both retinal endothelial and Müller cells to determine if these cells responded in a similar manner to whole rat retina.

## Research Design and Methods

### BBZDR/Wor Rat

Prior to initiation of the studies, all rat work was approved by the University of Tennessee Health Science Center Institutional Animal Care and Use Committee under protocol #1992. BBZDR/Wor lean (10 male) and obese rats (10 male) were purchased from Biomedical Research Models (Worcester, MA). Glucose measurements were taken weekly and once obese rats reached a glucose of >250mg/dl, an initial electroretinogram (ERG) was done prior to treatment initiation. Five lean and five obese rats were placed into treatment groups and received salicylate (27mg/kg/day) added to their feed daily for 2 months. This concentration was reported in other work to be in lower levels of intermediate therapy (~28-56mg/kg/day) range for humans taking aspirin salicylates (2-4g/day assuming a 70kg adult) [11]. After 2 months of treatment, animals in all four groups (lean, lean+sal, obese, obese+sal) underwent an additional ERG prior to sacrifice.

The BBZDR/Wor rat was produced using the BBZDP/Wor (BB rat with an *Iddm2* type 1 genetic locus) and mating with lean BBZ/Wor rats (BB rat with Zucker diabetic gene) to remove the *Iddm2* locus. The obese male BBZDR/Wor rat spontaneously develops type 2 diabetes at 10 weeks of age (100%) when fed standard rat chow [17,18]. The obese BBZDR/Wor rat is lymphopenic, hyperinsulinemic with peripheral insulin-resistance and develops spontaneous autoimmune noninsulin dependent diabetes mellitus at an age of 70 days [17]. The BBZDR/Wor rat model of type 2 diabetes has been used for studies of myogenic tone of the ophthalmic artery [19] and vascular damage repair using endothelial cell progenitors [20]. Results in the oxygen-induced retinopathy model and the STZ model using endothelial cell progenitor cells suggest that the BBZDR/Wor rat is a good model of type 2 diabetic retinal changes [20].

Sodium salicylate did not alter the intraocular pressure or blood glucose levels of the obese rats (Table 1). All animal weights and glucose measurements were made on animals of the same age.

**Table 1:** Body weight, glucose (mg/dl) and intraocular pressure (mmHg) for BBZDR/Wor lean and obese rats.

	Num	Body Weight (g)	Glucose	IOP(mmHg)
Lean	5	486.5±16.4	91.2±10.3	11.3±2.1
Lean+Sal	5	480.3±22.5	93.3±12.2	10.2±1.3
Obese	4	898±19.8*	583.5±40.7*	10.8±1.6
Obese+Sal	5	869±24.2*	519±32.9*	11.1±1.9

*P<0.05 vs. lean and lean+Sal. All parameters were measured on animals of the same age.

### Electroretinogram

Prior to treatment initiation and prior to sacrifice (2 months of treatment) for biochemical analyses, animals were subjected to ERG analyses to evaluate the changes in the electrical activity of the retina as we have done previously [2,21]. After dark adaptation overnight, ERG responses were recorded from both eyes using platinum wire corneal electrodes, forehead reference electrode, and ground electrode in the tail. Pupils were fully dilated using 1% tropicamide solution (Alcon, Ft. Worth, TX). Methylcellulose (Celluvise; Allergan, Irvine, CA) drops were applied as well to maintain a good electrical connection and body temperature was maintained at 37°C by a water-based heating pad. ERG waveforms were recorded with a bandwidth of 0.3-500Hz and sampled at 2kHz by a digital acquisition system and were analyzed using a custom-built program, which allowed a measurement of a-wave, b-wave and oscillatory potential from all animals (MatLab, Mathworks, Natick, MA). Statistics were done on the mean ±SD amplitudes of the a- and b- wave of each treatment group.

### Intraocular pressure

(IOP) was measured monthly using a tonometer (TonoLab, Colonial Medical Supply, Franconia, NH). Briefly, the tip of the probe of the tonometer was placed at the cornea of the eye. During measurements, the tip of the probe hit the cornea six times and gave the IOP reading of that eye. This procedure was carried out for both eyes.

### Retinal Endothelial Cells (REC)

Primary human retinal endothelial cells (REC) were obtained from Cell Systems Corporation (CSC, Kirkland, Washington) as we have done in the past [2,22]. Cells were grown in M131 medium containing microvascular growth supplements, 10μg/mL gentamycin, and 0.25μg/mL amphotericin B (Invitrogen, Carlsbad, CA) as we have done in the past [2]. Before the experiments, some cells were transferred to high glucose (25 mM) medium for 3 days or maintained in normal glucose (5 mM) medium throughout the growth phases (usually 3–5 days) and grown to 80% confluence. Primary cells (passage 4–6) were used. Growth was slowed in the cells by incubating cells in high or normal glucose medium without MVGS for 24 hours and then treated with 5–20 mM sodium salicylate for 2 hours as indicated.

### Müller cells

Rat retinal Müller cells (rMC-1, courtesy of Vijay Sarthy, Northwestern University) were grown in 5mM or 25mM glucose DMEM medium (HyClone Laboratories, Logan, UT). We chose to use this model as we have previously published the effects of β-adrenergic receptor agonists on insulin signaling in these cells [4]. Medium was supplemented with 10% FBS and antibiotics. Cells were cultured to 80% confluency (2–4 days), and then cells were starved for 18–24 hours by reduction to 2% FBS in the growth medium to eliminate any residual growth factors in the serum. We chose to reduce serum to 2% rather than complete starvation to eliminate activation of apoptotic pathways. Additionally, we have used this method in the past for measurements of TNFα and insulin pathways [4].

### Western Blotting

For isolation of the retina, the entire globe is removed from the animal at sacrifice. The cornea is cut and lens removed. The vitreous is removed with the lens and the retina is the off-white tissue remaining. The retina is placed into a tube with lysis buffer. After treatments, whole retinal lysates and REC and Müller cells were collected into lysis buffer containing protease and phosphatase inhibitors and processed for Western blotting. Equal amounts of protein from the cell extracts were separated on precast tris-glycine gels (Invitrogen) and blotted on the nitrocellulose membrane. After blocking in TBST (10 mM Tris-HCl buffer, pH 8.0; 150mM NaCl; 0.1% Tween 20) and 5% (wt/vol) BSA, the membranes were treated with appropriate primary antibodies followed by incubation with secondary antibodies labeled with horseradish peroxidase. Antigen-antibody complexes were detected by chemiluminescence reagent kit (Thermo Scientific, Waltham, MA). Primary antibodies used were β- actin (1:2000, Santa Cruz Biotechnology, Santa Cruz, CA) total and phosphorylated IκBα (Ser 32), total and phosphorylated IRS1 (Ser307), SOCS3, total and phosphorylated Akt (Ser 473), cytochrome c, Bax, Bcl-xL (1:500, Cell Signaling, Danvers, MA) and total and phosphorylated insulin receptor (Tyr 960), (1:500, Cell Applications, San Diego, CA). Densitometric analysis was carried out using Kodak Image Station 4000MM. Data is expressed as ratio of total proteins of interest to beta actin levels and the ratios were compared to control group for each individual experiment. In the case of phosphorylated proteins, data is expressed as a ratio of phosphorylated protein levels to total protein levels compared to control group.

### ELISA analysis

TNFα protein concentrations were measured in the cell lysate after salicylate treatment using a TNFα ELISA (ThermoFisher, Pittsburgh, PA) according to the manufacturer’s instructions. Equal protein levels were added to the ELISA to ensure cell numbers did not affect concentrations. A cleaved caspase 3 ELISA (Cell Sigaling, Danvers, MA) was used to measure levels of active apoptotic marker in retinal cells. Equal amount of protein was loaded for cleaved caspase 3 ELISA to allow for analysis using the optical density measurements.

### Statistics

All the experiments were repeated at least in triplicate. Data are presented as mean± standard error of mean. Data was analyzed with a Kruskal-Wallis test, followed by Dunn’s test with P values < 0.05 considered statistically significant. One representative blot is shown in case of western blots. Western blots densitometry is measured in arbitrary units (A.U.)

## Results

### Sodium salicylate significantly improved ERG amplitudes on BBZDR/Wor obese rats

**Fig 1:**
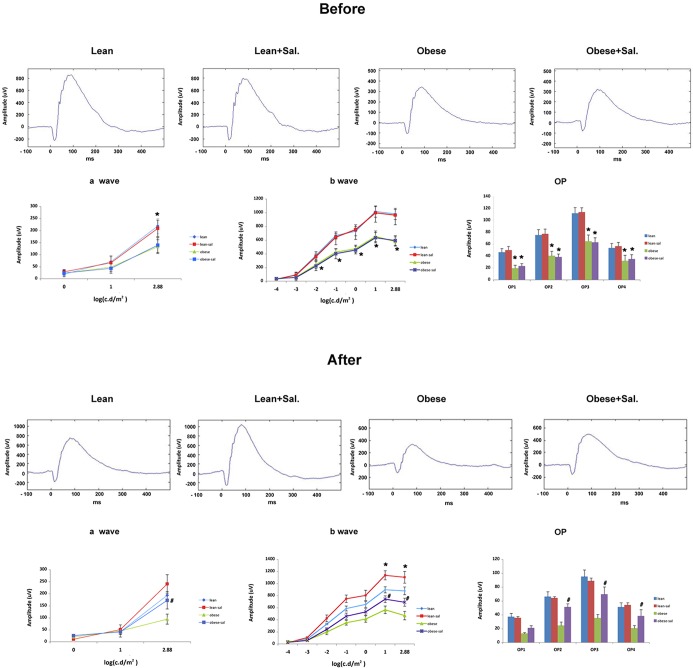
Electroretinogram analyses of BBZDR/Wor lean and obese rats shows that salicylate improved B-wave and oscillatory potentials. Top Panel shows ERG recordings before treatment and bottom panels represent ERG measurements after 2 months of treatment. a-wave (left), b-wave (middle), and oscillatory potentials (right) in lean, lean+salicylate (lean+sal), obese, obese+salicylate (obese+sal) are presented. *P<0.05 vs. lean, N = 5 per group. #P<0.05 vs. obese untreated. Data are mean ±SD.

Others had suggested that sodium salicylate was able to prevent reductions in neuronal thickness and formation of degenerate capillaries in a type 1 rodent model [11]. One of the goals of this work was to determine whether salicylate could improve retinal function in a type 2 diabetic model. Few changes were noted between lean and lean+Sal or obese vs. obese+Sal before treatment was initiated. The obese and obese+Sal rats were approximately 1 month older than lean rats because it took an extended period of time to develop diabetes. However, since our goal was to determine whether salicylate improved retinal function vs. untreated animals, the 1 month age difference should not matter since obese and obese+Sal were of the same age and had similar initial amplitudes. For lean rats, salicylate significantly increased the b-wave amplitudes after 2 months of salicylate treatment. The reason for salicylate increasing b-wave amplitudes in lean rats is unclear. Untreated obese rats have a significant reduction in the a-wave (at 2.88 intensity) and b-wave at all intensities when compared to lean rats (Fig 1). When obese rats were treated for 2 months with salicylate, a-wave (at 2.88 intensity) and b-wave ampltiudes returned to levels similar to untreated lean rats (Fig 1).

### TNFα and IRS-1^Ser307^ were significantly reduced in obese rat retina fed chow containing salicylate

**Fig 2:**
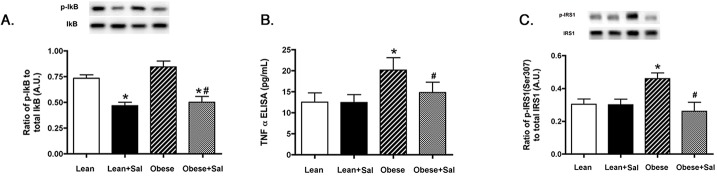
IkBα, TNFα and IRS-1^Ser307^ levels are all reduced in whole retinal lysates from lean and obese BBZDR/Wor rats by salicylate treatment. Western blotting for IkB (Panel A), ELISA results for TNFα (Panel B) and Western blot data for IRS-1^Ser307^ (Panel C) in lean, lean+salicylate (lean+sal), obese, obese+salicylate (obese+sal) BBZDR/Wor rat retina showing that salicylate treatment reduced IkBα, TNFα and IRS-1^Ser307^ in BBZDR/Wor obese rats and IkBα in lean rats. *P<0.05 vs. lean, #P<0.05 vs. obese. N = 5, data are mean±SEM.

We have previously demonstrated that TNFα is increased in rat retina with type 1 [2] and type 2 diabetes [23]. Using retinal cells cultured under hyperglycemic conditions, we have also demonstrated that increased TNFα levels led to increased apoptosis of REC [3] and Müller cells [24]. We first needed to ensure that phosphorylated IkB was reduced in the rat retina to verify that salicylate worked in the rats (Fig 2A). We then wanted to determine whether TNFα was increased in the BBZDR/Wor obese animals and whether salicylate could reduce TNFα levels. Fig 2B confirmed that TNFα is increased in the type 2 rat model, and demonstrated that salicylate was able to significantly reduce TNFα levels in these animals. Since TNFα can inhibit insulin signaling through increased phosphorylation of IRS-1 on serine 307 [5], we also measured IRS-1^Ser307^ levels in these animals. BBZDR/Wor obese rats, which received the salicylate-containing chow, had significantly reduced levels of IRS-1^Ser307^ compared to untreated obese rats (Fig 2C). Treatment with salicylate in lean rats significantly reduced IkB phosphorylation; however, salicylate had no effects on TNFα or IRS-1^Ser307^ in lean rat retina.

### Salicylate significantly reduced SOCS3 and IR^Tyr960^ in the retina of BBZDR/Wor obese rats

**Fig 3:**
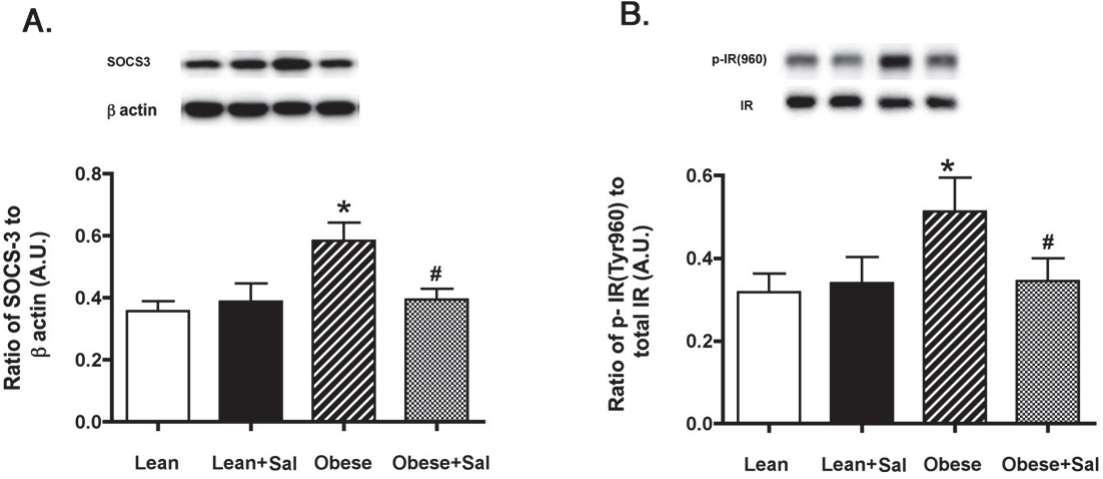
Salicylate reduced both SOCS3 and IR^Tyr960^ levels in rat retina from lean and obese BBZDR/Wor animals. Measurements were done in lean, lean+salicylate (lean+sal), obese, obese+salicylate (obese+sal) BBZDR/Wor rats. Salicylate treatment significantly reduced SOCS3 (Panel A) and IR^Tyr960^ phosphorylation (Panel B) in treated animals as measured by Western blotting. *P<0.05 vs. lean, #P<0.05 vs. obese. N = 5, data are mean±SEM.

In continuation of our work on TNFα-induced retinal insulin resistance, we measured SOCS3 and IR^Tyr960^ in the BBZDR/Wor lean and obese rats alone and treated with salicylate. Based on prior work demonstrating that TNFα activates SOCS3 [7] and SOCS3 can induce insulin resistance through activation of insulin receptor via phosphorylation on tyrosine 960 [8], we measured both SOCS3 and IR^Tyr960^ in the lean and obese animals. Fig 3A demonstrated that BBZDR/Wor obese rats treated with salicylate have reduced SOCS3 when compared to untreated obese animals. IR^Tyr960^ is also reduced following salicylate treatments, suggesting that reduction of IkB by salicylate can reduce insulin resistance in the type 2 rat model. Salicylate had no effects on lean animals.

### Pro-apoptotic markers, except caspase 3, are reduced in BBZDR/Wor obese rat retina fed with salicylate-containing chow

**Fig 4:**
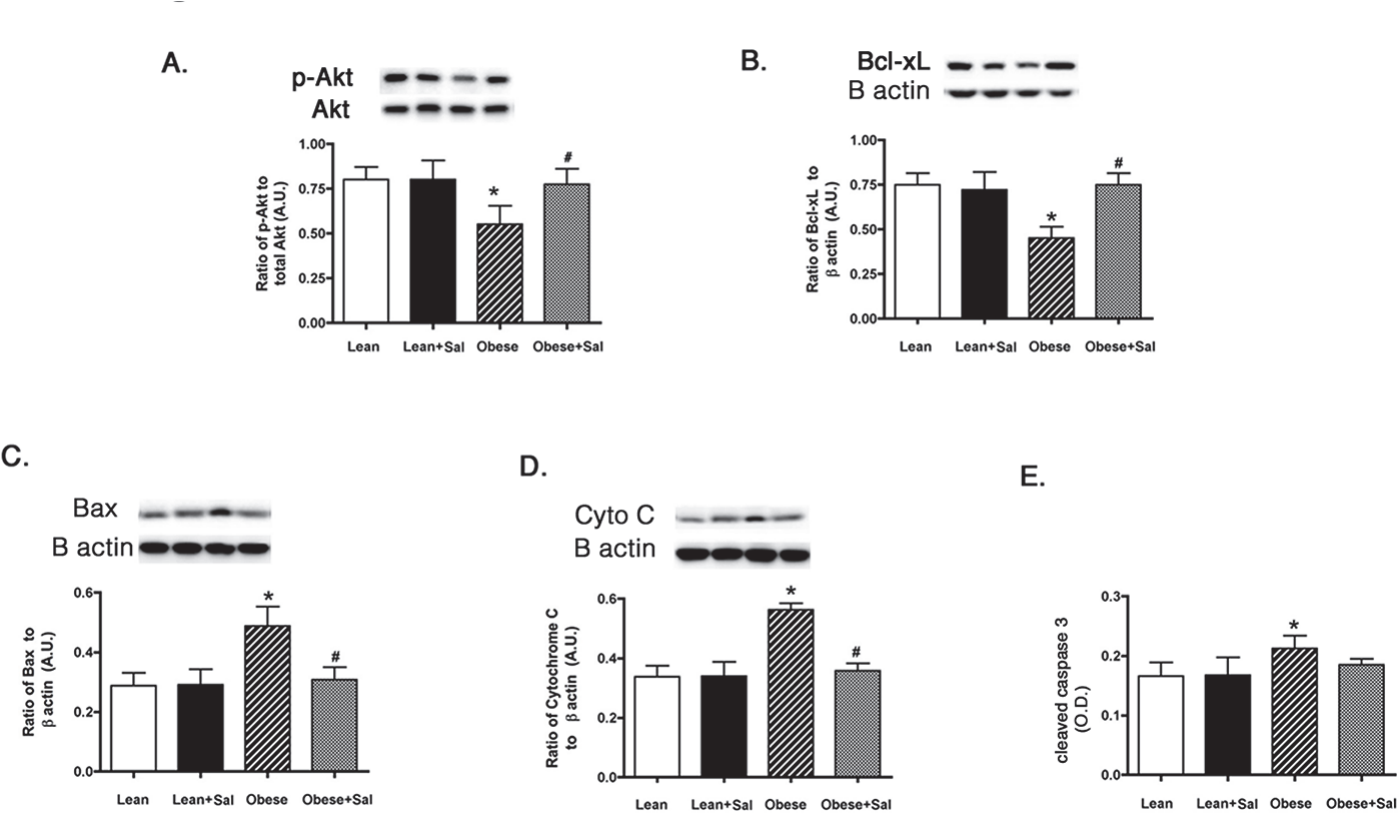
Anti-apoptotic markers are normalized in BBZDR/Wor obese rat retina treated with salicylate to levels seen in lean animals. Panels A, B show anti-apoptotic markers in lean, lean+salicylate (lean+sal), obese, obese+salicylate (obese+sal) BBZDR/Wor rats, while Panels C-E show pro-apoptotic markers from retinal lysates from the same lean and obese rats. *P<0.05 vs. lean, #P<0.05 vs. obese. N = 5, data are mean±SEM.

Since one major function of intact insulin signaling is to inhibit apoptosis, we wanted to measure key pro- and anti-inflammatory markers in the whole retinal lysates from the BBZDR/Wor lean and obese rats. Lean animals did not have any changes in apoptotic markers in response to salicylate chow. We found that salicylate significantly increased the serine (473) phosphorylation of the anti-apoptotic marker, Akt (Fig 4A) and Bcl-xL levels (Fig 4B) in the BBZDR/Wor obese animals. Additionally, salicylate reduced the levels of cytochrome C (Fig 4D) and Bax (Fig 4C). Salicylate had no effect on caspase 3 in these rat retinal lysates (Fig 4E). This likely may stem from salicylate actions on caspase 3, which appear to be cell-type and organ specific.

### Pro-apoptotic Markers are altered with Salicylate Treatment to REC and Müller cells in High Glucose

**Fig 5:**
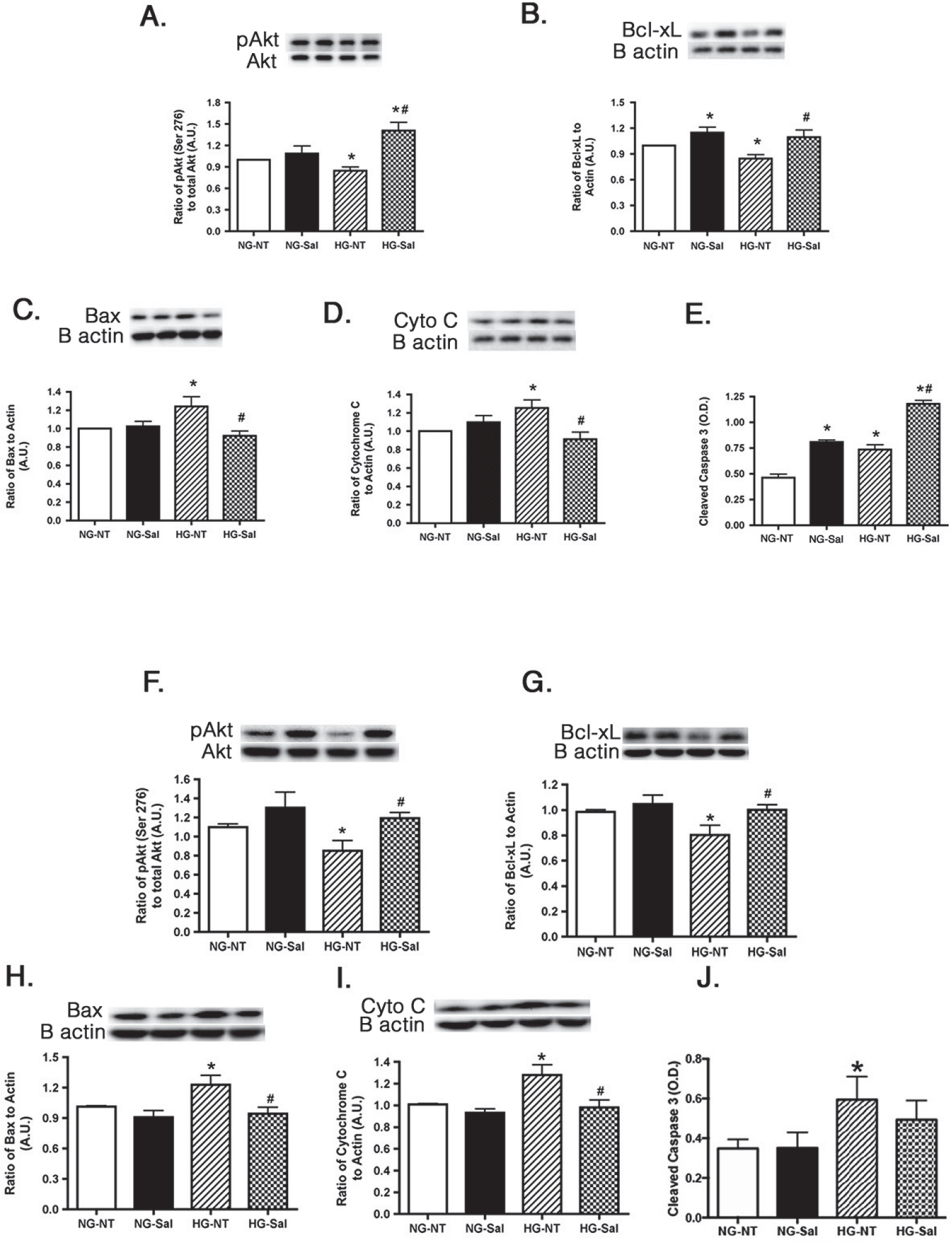
Apoptotic proteins are altered in REC and Müller cells cultured in high glucose and following salicylate treatment. The top two graphs are western blot results of anti-apoptotic proteins in normal glucose (NG) or high glucose (HG) and either untreated (NT) or treated with salicylate (Sal). Data is represented as ratio of phosphorylated Akt (Ser 473) to total Akt (A,F) and that of Bcl-xL to β-actin (B,G) compared to normal glucose untreated (NG-NT). The bottom three graphs show the proapoptotic proteins in cells after the same treatments. Western blots of Bax (C,H) and Cytochrome C (D,I) as compared to β-actin and ELISA results of cleaved caspase 3 (E,J) are shown. Panels A-E represent REC data, while panels F-J present Müller cell data. A representative blot is shown for each western blot. *P<0.05 vs. NG-NT; #P<0.05 vs. HG-NT. Data are mean± SEM. N = 4 for each group.

Retinas of the diabetic rats have increased levels of apoptotic markers [2]. Administration of sodium salicylate to the diabetic rats was reported to prevent diabetes-induced degeneration of retinal capillaries and neurons [11]. To evaluate whether salicylate ameliorated apoptosis in specific retinal cell types, REC and Müller cells, we measured key anti-apoptotic (Akt, Bcl-xL) and pro-apoptotic (Bax, cytochrome C, cleaved caspase 3) proteins in each cell type cultured in normal and high glucose. High glucose decreased the anti-apoptotic proteins in both cell types, with treatment of 20mM salicylate significantly increasing levels of Akt and Bcl-xL (Fig 5A and 5F and 5B and 5G). It is unclear why salicylate increased Bcl-xL levels in REC cultured in normal glucose. No other pro- or anti-apoptotic marker was altered in response to salicylate in cells cultured under normal glucose conditions. Salicylate was also effective in reducing the increased levels of Bax and cytochrome c in both cell types cultured in high glucose (Fig 5C and 5H and 5D and 5I). We found that cleaved caspase 3 levels were elevated by salicylate treatment in REC cultured in both normal and high glucose (Fig 5E and 5J). Salicylate has been reported to activate caspase 3 in other models, including the cochlea [16] and colorectal cancer cells [15]. The increase in activation of caspase 3 following salicylate treatment is in contrast to the observed changes of other pro-apoptotic proteins in high glucose. However, salicylate did decrease cleaved caspase 3 in retinal Müller cells. Data suggest that salicylate actions on caspase 3 may be cell-type and model specific.

### Sodium Salicylate Significantly Decreases IκBα Phosphorylation in both REC and Müller cells cultured in High Glucose

**Fig 6:**
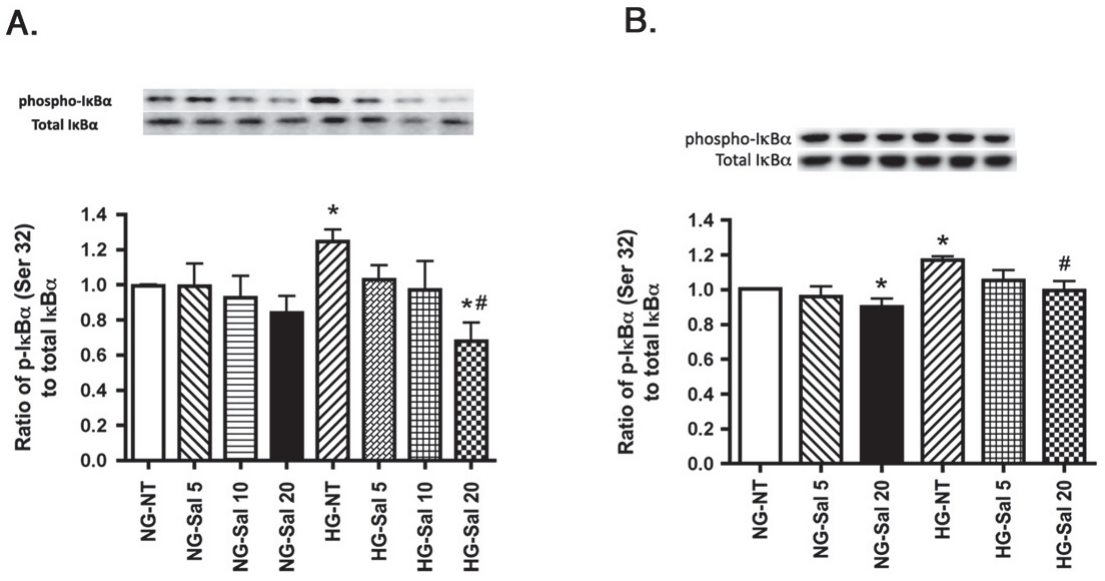
Sodium salicylate inhibits high glucose induced IκBα phosphorylation in both REC and Müller cells. Western blots results of phosphorylated IκBα are shown in normal glucose (NG) and high glucose (HG) in cells left untreated (NT) or treated with 5 mM salicylate (Sal 5), 10 mM salicylate (Sal 10) and 20 mM salicylate (Sal 20). Panel A represents REC data, while panel B shows Müller cells data. Data is presented as ratio of phosphorylated IκBα to total IκBα, compared to normal glucose untreated (NG-NT). A representative blot is given for Western blot result. *P<0.05 vs. NG-NT; #P<0.05 vs. HG-NT. Data are mean± SEM. N = 4 for each group.

Sodium salicylate is used to treat inflammatory diseases and improve insulin sensitivity [25]. High concentrations of sodium salicylate are known to mediate its anti-inflammatory property by inhibiting the cellular kinase IKK-β, as opposed to working through its classical target cyclooxygenase (COXs) [26]. IκB is phosphorylated by IKK-β that leads to its degradation and activation of NF-κB [27]. First, we wanted to determine the most effective concentration of salicylate that decreases IκB phosphorylation in REC and Müller cells. We treated the cells with increasing concentrations of sodium salicylate from 5 mM to 20 mM for 2 hours both in normal and high glucose medium. REC and Müller cells cultured in high glucose medium had increased phosphorylation of IκB. Treatment with 20mM sodium salicylate significantly reduced phosphorylation of IκB in high glucose as compared to 5 or 10 mM salicylate treatment (Fig 6A and 6B). This concentration of salicylate was thus chosen for all the subsequent experiments. 20mM salicylate also decreased IkB phosphorylation in retinal Müller cells cultured in normal glucose.

### Salicylate Treatment Reduces TNFα and Phospho-IRS-1^Ser307^ in cells cultured in High Glucose

**Fig 7:**
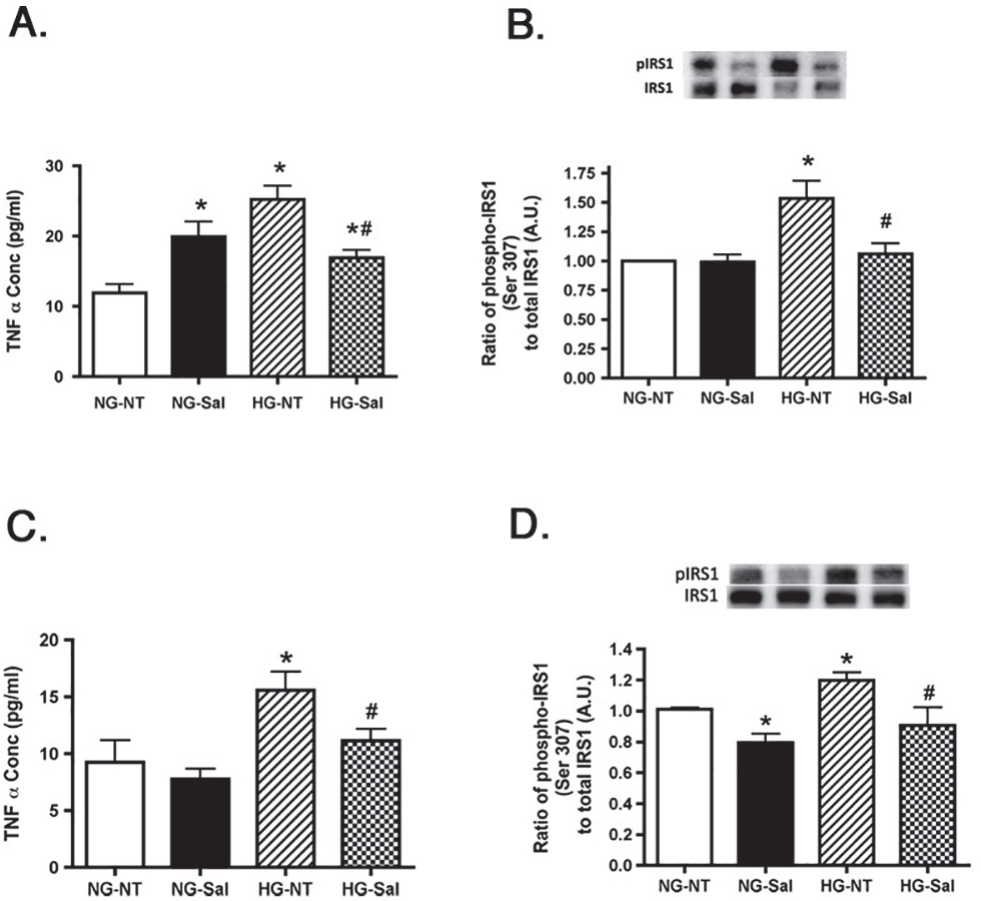
TNFα and IRS-1^Ser307^ are reduced after salicylate treatment in both REC and Müller cells cultured in high glucose. Panel A,C is the TNFα concentration in REC in normal glucose (NG) or high glucose (HG) and either untreated (NT) or treated with salicylate (Sal). Panel B,D is the ratio of phosphorylated IRS-1^Ser307^ to total IRS-1 after the same treatments. Panels A and B show REC data, while panels C and D present Müller cell data. A representative blot is given for IRS-1 western blots. *P<0.05 vs. NG-NT; #P<0.05 vs. HG-NT. Data are mean± SEM. N = 4 for each group.

We have previously reported that REC and Müller cells cultured in high glucose had higher amount of TNFα in the lysate compared to lysate from cells cultured in normal glucose. High amounts of TNFα cause an increase in phosphorylation of IRS-1^Ser307^ leading to insulin resistance and apoptosis [3,4]. To determine whether the effective concentration of salicylate in cell lysates was also able to affect this pathway, we assessed the levels of TNFα and phospho-IRS-1^Ser307^ in the cell lysates. As before, we saw an increase in TNFα and phosphorylated IRS-1^Ser307^ when the cells were cultured in high glucose. Upon treatment of cells with 20mM salicylate, TNFα levels were significantly reduced in high glucose medium in REC (Fig 7A) and Müller cells (Fig 7C). Treatment of REC in normal glucose with salicylate significantly increased TNFα levels (Fig 7A). While unexpected in this model, increased TNFα following salicylate has been reported in models of tinnitus [28,29]. Salicylate treatment also reduced the elevated phospho-IRS-1^Ser307^ in REC cultured under high glucose conditions and in Müller cells in both normal and high glucose (Fig 7B and 7D), likely through the reduced TNFα levels.

### Increased SOCS3 and Phospho-IR^Tyr960^ Levels in High Glucose are returned to baseline by Salicylate treatment

**Fig 8:**
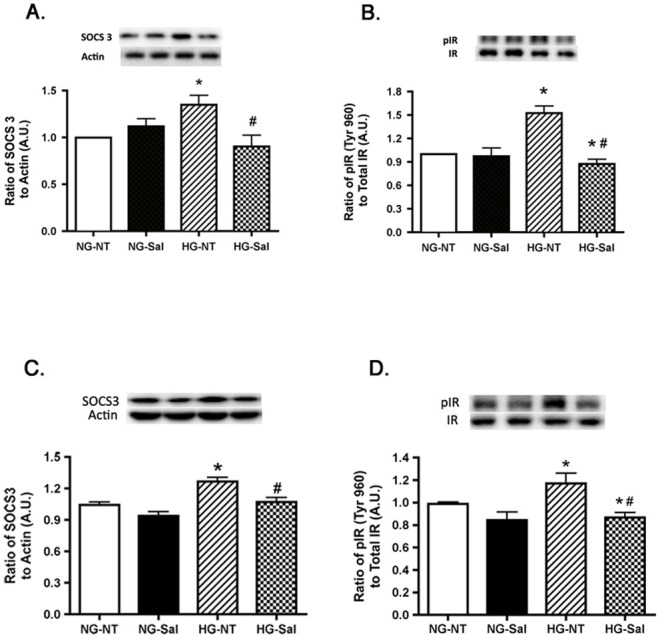
Salicylate reduces high glucose-induced SOCS3 and phosphorylated IR^Tyr960^ in both REC and Müller cells. Western blot results are shown in panel A for SOCS3 in normal glucose (NG) or high glucose (HG) and either untreated (NT) or treated with salicylate (Sal). Panel B is the ratio of phosphorylated IR^Tyr960^ to total IR on cells after salicylate treatment. Panels A,B are REC data; Panels C,D are Müller cell data. A representative blot is given for each western blot. *P<0.05 vs. NG-NT; #P<0.05 vs. HG-NT. Data are mean± SEM. N = 4 for each group.

TNFα causes an increase in SOCS3 levels in both REC and Müller cells, with SOCS3 inducing insulin resistance through phosphorylation of insulin receptor at tyrosine 960. Phosphorylation of IR^Tyr960^ blocks the interactions between IRS-1 and insulin receptor, such that insulin receptor cannot signal to Akt [30]. SOCS3 and IR^Tyr960^ were both elevated in REC and Müller cells cultured in high glucose and salicylate was found to significantly reduce the levels of SOCS3 and phospho-IR^Tyr960^ to levels similar to normal glucose (Fig 8A and 8C and 8B and 8D), thus suggesting that salicylate improved insulin signaling through reduction of both the TNFα and SOCS3 pathways. Salicylate did not alter SOCS3 levels or the phosphorylation of IR^Tyr960^ in either cell type cultured in normal glucose.

## Discussion

Previous work had demonstrated that sodium salicylate was effective in reducing many histological markers of diabetic retinopathy in a type 1 diabetic rat model [11]. The overall goal of this study was two-fold; we wanted to demonstrate that salicylate would also improve retinal function (as measured by ERG) in a type 2 diabetic rat model, as well as provide a potential mechanism of action for the improved retinal function after salicylate treatment using analyses of insulin signaling in both salicylate treated animals, as well as in retinal endothelial cells and Müller cells. Salicylate is also reported to be effective in a rat model of Parkinson’s disease through its anti-inflammatory actions [12]. Our data in the BBZDR/Wor obese rats demonstrated that 2 months of salicylate treatment in rat chow was effective in preventing the loss of b-wave and oscillatory potential amplitudes when compared to untreated animals. Amplitudes of obese+salicylate rats were similar to levels observed in untreated lean rats, despite having significantly higher body weight and glucose levels. Salicylate also significantly increased b-wave amplitudes in lean rats. Thus, it appears that salicylate is effective in improving retinal function in a type 2 diabetic rat models.

The second goal of this study was to determine a potential mechanism for the improvement in the retinal function in the BBZDR/Wor obese rats. Sodium salicylate can decrease IkB in the diabetic rat retina [31]. We have previously reported increased TNFα and IRS-1^Ser307^ levels in the BBZDR/Wor obese rats [23], as well as in REC [3] and Müller cells [32] cultured in high glucose conditions. Because of our previous work on IRS-1^Ser307^ and the link between IRS-1^Ser307^ and TNFα [5], we chose to focus on IRS-1 instead of IRS-2. Others have also reported that drugs, such as HIV protease inhibitors, also work to induce insulin resistance in Chinese hamster ovary cells and adipocytes through the reduction in phosphorylation of IRS-1 on serine 307 [9]. Similarly, work in Wistar rats fed a high fatty acid diet demonstrated increased levels of IRS-1^Ser307^, which was reduced after salicylate treatment [14]. In addition to IRS-1^Ser307^, salicylate actions on TNFα provide a clue as to antioxidant actions of salicylate. Derivatives of salicylate have been reported to reduce iNOS and COX2 through reduced NFkB signaling in rat prostate endothelial cells [33]. Additionally, salicylate reduced oxidant levels in a rodent model of parkinsonism [34]. Work in *C*. *elegans* also showed that salicylate can delay age-induced decreases in motility and extend the lifespan due to increased antioxidant levels and reduced levels of reactive oxygen species [35]. Others reported that salicylate can prevent free fatty acid-induced insulin resistance through increased superoxide dismutase levels and reduced iNOS in the liver [36]. In work in coronary arteries from diabetic mice, Yang et al reported that salicylate reduced the TNFα/NFkB interaction, leading to reduced oxidative stress, thus preserving normal vasodilation [37]. In contrast, others have reported that the actions of salicylate work through anti-inflammatory pathways rather than anti-oxidant pathways in a chronic parkinson’s model [12], which agrees with the finding of reduced iNOS reported by He [36]. It is highly likely that salicylate works to both reduce inflammation and oxidative stress, both of which would improve insulin signaling in the retina. Additional studies will be directed at dissecting salicylate actions as an antioxidant vs. anti-inflammatory in the retina.

In addition to the actions on TNFα, salicylate also reduced SOCS3 levels, again providing a mechanism of improved insulin signaling observed in the BBZDR/Wor obese rats. Similarly in a SOCS3 cKO mouse model, salicylate partially improved insulin resistance [38]. The HIV protease inhibitors also had reduced SOCS1 and SOCS3 levels [9]. SOCS3 induces insulin resistance through increased phosphorylation of the insulin receptor on tyrosine 960, which inhibits the insulin receptor/IRS-1 interaction [8]. As expected, salicylate also reduced phosphorylation of IR^Tyr960^, leading to increased Akt activity.

The data clearly demonstrate that salicylate can improve insulin signaling through reduced TNFα and SOCS3 levels, both in rat retina, as well as in REC and Müller cells. An interesting finding from this work is that salicylate reduced all pro-apoptotic markers, except caspase 3. In previous work with pioglitazone, a novel β-adrenergic receptor agonist, Compound 49b, and in a blast-induced injury model, the drug of interest reduced all pro-apoptotic markers studied (Bax, cytochrome C, and caspase 3) [2,23,39]. Salicylate appears to have unique effects on caspases, as it is reported to induce apoptosis in myeloid leukemia cells lines [40], in colorectal cancer cells [15], and in guinea pig cochlea [16]. However, salicylate reduced TUNEL labeling in a type 1 diabetic rat model [11] and in a model of paraquat-induced apoptosis of the lung [41]. In our data, salicylate increased protein levels of the anti-apoptotic markers, Akt and Bcl-xL, while decreasing Bax and cytochrome C. Salicylate either had no effect in Müller cells and rat retina or increased cleaved caspase 3 levels in REC. Therefore, it appears that the retina is another cell type that responds to salicylate in different ways for cell types. In addition to caspase 3, salicylate significantly increased TNFα in REC, but not in Müller cells cultured in normal glucose. Others have reported that salicylate can increase TNFα levels in models of tinnitus [28,29]. Future work can be designed to tease out the actions of salicylate on TNFα and caspase 3 in the retina and retinal cells under specific glucose conditions.

Taken together, it is clear that salicylate was able to improve retinal function likely through improved insulin signaling in the retina in a type 2 diabetic rat model and retinal cells. Salicylate significantly reduced TNFα and SOCS3 levels, leading to increased levels of anti-apoptotic proteins. These data suggest that the effectiveness of salicylate in improving retinal markers of diabetic retinopathy can be extended to type 2 diabetes.
